# Increasing the potential for malaria elimination by targeting zoophilic vectors

**DOI:** 10.1038/srep40551

**Published:** 2017-01-16

**Authors:** Jessica L. Waite, Sunita Swain, Penelope A. Lynch, S. K. Sharma, Mohammed Asrarul Haque, Jacqui Montgomery, Matthew B. Thomas

**Affiliations:** 1 Center for Infectious Disease Dynamics and Department of Entomology, Pennsylvania State University, University Park, PA, 16802, USA; 2 Jigyansha, International Center of Excellence for Malaria Research, Sector 1, Rourkela, Odisha, India; 3 University of Exeter, College of Life & Environmental Sciences, Penryn Campus, Cornwall, TR10 9FE, United Kingdom; 4 National Institute of Malaria Research (ICMR), Sector 8, Dwarka, New Delhi-110 077, India; 5 Eliminate Dengue Program, Monash University, Clayton, Victoria 3800, AUS

## Abstract

Countries in the Asia Pacific region aim to eliminate malaria by 2030. A cornerstone of malaria elimination is the effective management of *Anopheles* mosquito vectors. Current control tools such as insecticide treated nets or indoor residual sprays target mosquitoes in human dwellings. We find in a high transmission region in India, malaria vector populations show a high propensity to feed on livestock (cattle) and rest in outdoor structures such as cattle shelters. We also find evidence for a shift in vector species complex towards increased zoophilic behavior in recent years. Using a malaria transmission model we demonstrate that in such regions dominated by zoophilic vectors, existing vector control tactics will be insufficient to achieve elimination, even if maximized. However, by increasing mortality in the zoophilic cycle, the elimination threshold can be reached. Current national vector control policy in India restricts use of residual insecticide sprays to domestic dwellings. Our study suggests substantial benefits of extending the approach to treatment of cattle sheds, or deploying other tactics that target zoophilic behavior. Optimizing use of existing tools will be essential to achieving the ambitious 2030 elimination target.

The WHO Roll Back Malaria (RBM) partnership recently produced an updated version of the Global Malaria Action Plan for 2016–2030 (GMAP2)^1^. This plan aims to decrease malaria cases globally by 90% compared to 2015 levels and eliminate malaria in at least 35 additional countries, by 2030. Inter-country alliances have formed to work together towards the goal of regional endemic malaria elimination by 2030, with one of the largest being the Asia Pacific Malaria Elimination Network (APMEN). India joined APMEN in 2015; having the most malaria cases in the region, elimination of malaria in India is central to the broader success of APMEN.

Currently, strategies to control and ultimately eliminate malaria rely heavily on the broad-scale implementation of insecticide-based interventions, including indoor residual spraying (IRS) and insecticide treated nets (ITNs), targeting the adult mosquito vectors. While IRS and ITNs effectively target mosquitoes that bite humans (anthropophilic) and that feed and rest indoors (endophagic and endophilic), numerous malaria vectors exhibit alternative feeding and resting behaviors. Human blood indices (the percentage of mosquitoes sampled that are positive for human blood) for mosquito species that transmit malaria are rarely 100%, indicating most mosquito populations feed on other vertebrates^2^, most often cattle^3^. In some settings, non-human feeding (zoophagy), together with outdoor resting in structures such as in cattle sheds (exophily), are the predominant behaviors.

Zoophilic vectors are important in many regions of the world. *An. arabiensis* (parts of Africa), *An. albimanus* (Latin America region), and *An. sinensis* (Asia-Pacific region) are all key malaria vectors, yet exhibit strong zoophilic and exophilic behaviors^4^,^5^,^6^. That zoophilic vectors can be so important for malaria transmission might seem counterintuitive since *Plasmodium* parasites responsible for human malaria cannot infect cattle, so bites taken on cattle instead of humans represent reduced opportunities for vectors to acquire or transmit *Plasmodium*^7^,^8^,^9^. However, livestock kept in close proximity to humans can support higher transmission by attracting mosquitoes into areas where they will encounter and feed on human hosts opportunistically (zoopotentiation)^7^,^10^.

In India, the vector species complexes responsible for the majority of malaria transmission (*Anopheles culicifacies, An. fluviatilis,* and *An. stephensi*) are primarily zoophilic^11^,^12^,^13^, feeding much more on cattle than humans^14^. However, the degree of zoophily and exophily varies by species type (these species occur as species complexes) and the development of optimal control tactics depends on understanding the specific feeding and resting behavior of the species complexes within particular locations.

Our study took place in Odisha state in India, where nearly half of all *Plasmodium falciparum* cases in India and over a third of all deaths due to malaria occur, even though only 4% of the population resides in this state^15^. Sundergarh district in Odisha has a long history of high malaria incidence^15^,^16^. From 2005–2010, the annual parasite incidence (API, which is the number of clinical cases per 1000 people per year) in the district ranged from 17.84 to 23.31 (*Source*: Data from the Office of the Chief District Medical Officer, Sundergarh). Earlier studies of malaria mosquitoes in this district indicate there are two key species complexes responsible for transmission. *Anopheles culicifacies* (a mix of B, C, and E types, which are largely zoophilic) maintains low level malaria transmission year round and drives a peak in transmission during the monsoon season when mosquito numbers increase^15^,^17^. Classically, there is then a second peak in transmission driven by a short-term seasonal upsurge in anthropophilic *An. fluviatilis* (S-type) during the retreating monsoon period^15^,^17^.

Whether present-day species complexes and feeding behaviors match these historical patterns is unknown. In the current study, therefore, we re-examined the role of the *An. culicifacies* and *An. fluviatilis* sibling species types in malaria transmission across 6 study villages in Sundergarh district. For both species we observed adult mosquitoes to rest predominantly in cattle sheds and exhibit a high level of cattle feeding (33–35% blood meals identified as bovine only), although mixed feeds on humans and livestock were common (40–45% mixed blood meals). Consistent with this feeding and resting behavior, we found *Anopheles culicifacies* to be a mix of zoophilic B, C, and E types. We also found an unexpected shift in *An. fluviatilis* from the anthropophilic S-type to zoophilic T-type, compared with historical patterns. Based on these observations, we developed a basic malaria transmission model to explore the likely effectiveness of IRS and ITNs in this location. We show that when zoophilic vectors dominate transmission, the classic approaches of IRS and ITNs will be insufficient to achieve elimination, even if implemented at maximal achievable coverage. However, we also show that directing even modest amounts of effort to specifically increase mosquito mortality associated with zoophilic behavior can shift the balance towards elimination.

## Methods

### Study area

The main entomological study was carried out in 6 villages near the malaria endemic city of Rourkela in Sundergarh district from October 2013 to September 2014. A further follow-up study designed to confirm the observations from the initial survey was carried out in 9 new additional villages in this district during October 2014–February 2015. Rourkela is an industrial city of Sundergarh district in Odisha state and is located in the Garhjat hills of eastern plateau between 20°-12′N and 84°-53′E at an altitude of 200 m above sea level. The study villages were representative of rural villages within 40 kilometers of Rourkela and matched for habitat types (forested hills, rocky streams, rivers, springs and paddy fields in the valleys). The mean minimum temperature during the coldest month was 14.3 °C, and the mean maximum temperature during the warmest month was 39.2 °C in the study area. Average annual rainfall ranges 160–200 cm with most rain associated with the Southwest monsoon (June–September) and retreating Northeast monsoon (December–January). IRS spraying of human dwellings with either DDT or synthetic pyrethroids is done twice yearly in this region as per the NVBDCP policy in locations with an annual parasite index (i.e. malaria cases per 1000 people per year) of 2 or above^18^.

### Mosquito collection

Indoor resting mosquitoes were collected monthly between 0600 to 0800 h in each village from 4 human dwellings and 4 cattle sheds that were initially randomly selected and then re-visited each month. An extra *An. fluviatilis* collection was done Oct. 2014–Feb. 2015 in random villages to confirm species composition in a second season (a total of 7 houses and 22 cattle sheds visited across 9 villages). Mosquitoes were collected for 15 min each from each structure using a mouth aspirator and flashlight, with equal time spent collecting from eaves, walls and roofs. Mosquitoes were brought to the laboratory for processing and taxonomic identification.

### Species and type identification, blood meal identification, and vector incrimination

Half-gravid females of *An. culicifacies* and *An. fluviatilis* identified morphologically^19^,^20^ were used for identification of sibling species and blood meal source. Ovaries of half-gravid females were removed and fixed in modified Carnoy’s fixative (1:3 acetic acid: methanol). From the same mosquito, blood from the gut was smeared on Whatman No. I filter paper (Whatman International Inc., Maidstone, England) for a blood meal assay. Head and thorax were separated and stored in microfuge tubes containing silica gel as a preservative for detection of sporozoites in the salivary glands. DNA samples from *An. fluviatilis*^21^ and *An. culicifacies*^22^ were identified to sibling species within each complex by using species-specific PCR assays.

Gravid and unfed *An. fluviatilis* were stored individually in 1.5 ml Eppendorf tubes filled with isopropanol after separating the head and thorax for PCR. Individually stored body parts of these mosquitoes were used for isolation of genomic DNA with a Qiagen Kit. Midgut blood smears of *An. fluviatilis* and *An. culicifacies* specimens identified to sibling species were subjected to blood meal source identification by PCR using an established protocol^23^. Homogenates of head and thorax from individual mosquitoes identified at sibling species level were processed for sporozoite detection of *P. vivax* and *P. falciparum* in the salivary glands by PCR^24^.

### Modeling malaria transmission and control

The model we used to extend our field and molecular results derives from the longstanding Ross-MacDonald framework used to define the Basic Reproductive Number (*R*_*0*_) of malaria^25^. *R*_*0*_ provides a measure of transmission intensity describing the number of new infections deriving from one primary infection. This framework is particularly relevant in the context of the current study as it enables us to determine the criteria for elimination (i.e. when *R*_*0*_ < 1).

The model is based on a standard expression for *R*_*0*_ see ref. 26, pages 399–400.


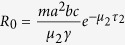


Parameters or variable definitions are given in Table 1.

#### Key model assumptions

1. The model considers as its baseline a vector population subject to lethal interventions applied via human dwellings and explores the effects of varying the probabilities of taking a human feed and the effects of adding cattle shed-based interventions.
2. Individual vectors are assumed to bite both human and livestock hosts with a given probability per feeding cycle of selecting a human host.
3. Vectors are assumed to feed on a single host type per feeding cycle.
4. Vectors are only affected by interventions applied to the location of their chosen host in each feeding cycle.
5. Vector mortality and host choice is assumed to be unaffected by vector age.
6. Once infectious, vectors do not recover and become non-infectious.
7. Juvenile density dependence effects mean that changes to the adult vector population size do not affect the number of newly-mature adults joining the population per day.
8. The human population size is not changed as a result of the interventions being considered.

We revised the standard *R*_*0*_ expression by expanding and substituting expressions described in Supplementary Materials (see Supplementary Materials, S1 model derivation and S2 model explanation) to give the following model:


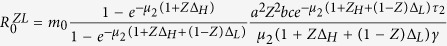


To explore the effect of varying assumptions about zoophagy, we assume an observed number of bites per person per day, *m*_*z*_*a* and calculate the implied value of the total population, *m*_0_, according to our assumptions about human-feeding probability, Z, as 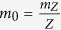.

Baseline parameter values used in the analysis are; *m*_*Z*_ = 30, *a* = 0.33, *c* = 0.8, *μ*_*2*_ = 0.131 (10% mortality per day plus 10% baseline feeding-related mortality), *b* = 0.1, *γ* = 0.0035, *τ* = 12 days. *a* and *μ*_*2*_ reflect an implicit assumption of a 3-day gonotrophic cycle length. Note that we ran sensitivity analysis around these base case values for all parameters and while the quantitative results changed, the qualitative conclusions were robust.

## Results

**Figure 1:**
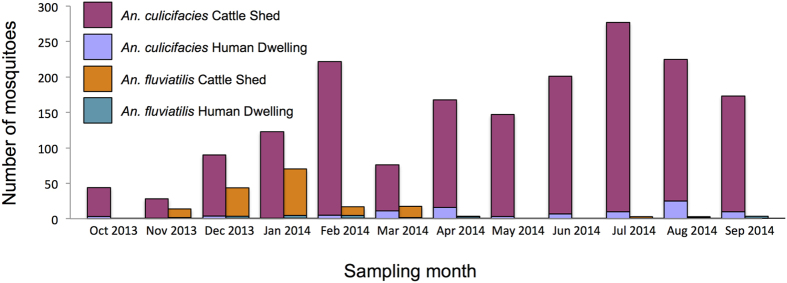
Monthly indoor resting density of *An. culicifacies* and *An. fluviatilis* collected from either cattle shed or human dwellings in Bisra and Birkera PHC areas from October 2013–September 2014.

Across all study sites, a total of 1774 *An. culicifacies* s.l. and 169 *An. fluviatilis* were collected between October 2013 and September 2014. The highest anopheline densities were observed during the advancing and retreating monsoons from July to September and October to January, respectively (Fig. 1 and Supplementary Materials, S3 environmental data). As in previous studies, *An. culicifacies* was observed year round, while *An. fluviatilis* was constrained to a 2–3 month breeding season. For both species, greater densities were found in cattle sheds compared with human dwellings (Fig. 1 and Table 2).

The sibling species type composition revealed by PCR is given in Table 2. Of the total *An. culicifacies* sampled, 99.8% were a mix of B, C, and E types. It is difficult to further distinguish B, C and E types with available assays, thus we will later refer to this group as “BCE type”. However, nearly 95% of the *An. culicifacies* were found in cattle sheds, suggesting predominantly types B and C since these are known to be zoophilic and the more anthropophilic E-type is rare in India^27^,^28^. *Anopheles culicifacies* A and D-type were encountered very infrequently (0.2% of the species total), and it is difficult to define their resting preferences with confidence based on the small samples (Table 2).

Consistent with earlier studies^15^,^17^, the *An. fluviatilis* species complex comprised types S and T only (no U and V-types were found). However, unlike the earlier studies, T-type was numerically dominant in the study villages (86.4% T type, and 13.6% S type). This general pattern was further confirmed in the follow-up survey conducted over 9 new additional villages that included a total of 7 houses and 22 cattle sheds, which showed 75.3% T-type and 24.7% S-type. In all cases, the majority of both S and T-types were collected from cattle sheds (Table 2).

### Host feeding behavior and sporozoite detection

**Figure 2:**
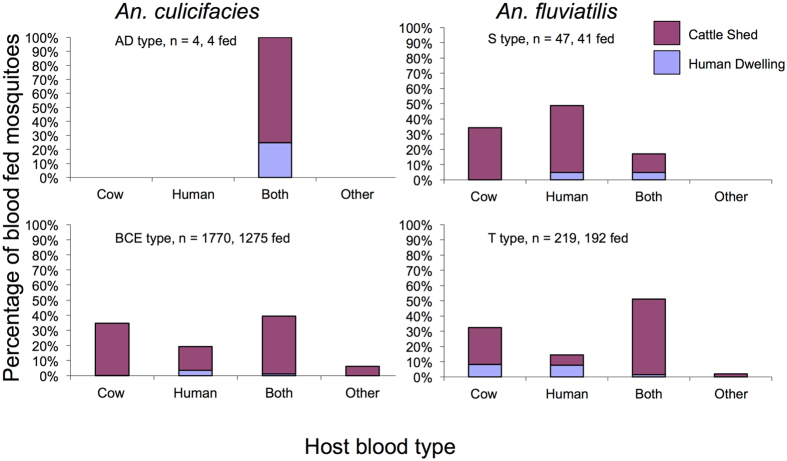
Mosquitoes that had blood fed separated by mosquito sibling species types. Shown is the proportion of each host blood meal type collected by resting location (cattle shed in red, human dwelling in purple). Left column is *An. culicifacies* (AD-type above, BCE-type below), and right column is *An. fluviatilis* (S-type above, T-type below) using combined data from collections 1 and 2 for *An. fluviatilis*.

Blood meal source was determined by PCR in individual *An. culicifacies* identified to sibling species type (Fig. 2). Of the 4 AD samples (0.2% of the total *An. culicifacies* collected), all were positive for mixed meals of bovine and human blood (Fig. 2 and Supplementary Materials, Supplementary Table S4), but none were positive for *P. falciparum.* The BCE types showed no clear feeding preferences, whether mosquitoes were collected from human dwellings or cattle sheds. Of *An. culicifacies* mosquitoes that fed on humans or cows, 37% had fed on cows only, 20.6% had fed on humans only, and 42.4% had mixed cow-human blood meals (Fig. 2 and Supplementary Material, Supplementary Table S4). From all 1770 samples of *An. culicifacies* BCE examined for malaria parasites, only 2 samples were positive for sporozoites (sporozoite positivity of 0.12%).

Blood mean analysis of *An. fluviatilis* provided limited evidence for exclusive human feeding (even the anthropophilic S-type) with high amounts of cow feeding (32.6% cow only) and mixed cow-human feeding (45%) overall for both types across all blood fed *An. fluviatilis* collected across the two sampling periods, regardless of whether mosquitoes were sampled from cattle sheds or human dwellings (Fig. 2 and Supplementary Material, Supplementary Table S4). No *An. fluviatilis* were found positive for *Plasmodium*.

### Model results

**Figure 3:**
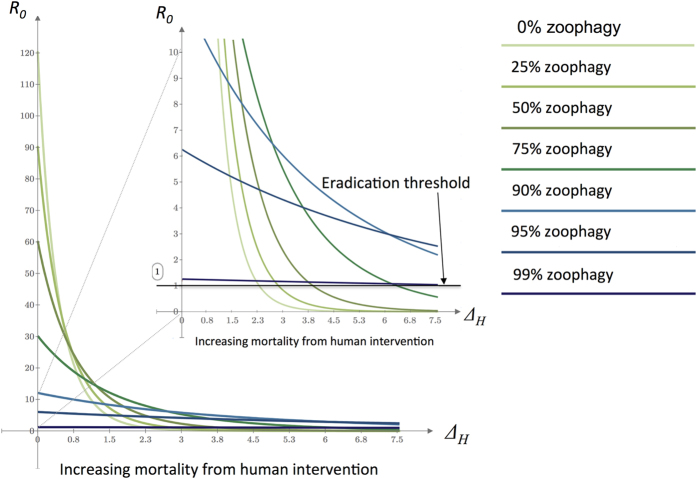
Increasingly zoophilic mosquito populations (less human feeding) make it harder to eliminate malaria (*R*_*0*_ < 1) with interventions that mosquitoes only encounter when attempting to feed on human blood. Mosquitoes here can bite either human or non-human hosts at each feed, and the percent that choose to bite humans is determined by mosquito preference. Inset shows an expanded view close to elimination threshold.

Our assumption that the mosquitoes responsible for malaria parasite transmission likely bite livestock as well as humans is borne out by our field data (Figs 1 and 2). In the absence of human-related interventions, the baseline *R*_*0*_ decreases with increasing zoophily since blood feeds on cows do not contribute directly to malaria transmission (Fig. 3 at 0% incremental mortality). When interventions that affect mosquito mortality associated with feeding on human hosts are added (e.g. IRS and ITNs), we find that as expected, *R*_*0*_ decreases with increasing levels of incremental mortality (analogous to increasing levels of coverage). However, the rate of decrease in *R*_*0*_ diminishes as the extent of zoophily increases. As a result, transmission can be higher with zoophilic mosquitoes than anthropophilic mosquitoes when conventional control tools are implemented at high coverage (Fig. 3).

Put simply, if all feeds are on humans, there is the possibility for human-based control measures to kill a mosquito at each of the multiple feeds between becoming infected and being able to deliver an infectious bite, but if most feeds are on cows, although the initial probability of becoming infected is low, the mosquito is unlikely to encounter control measures and more likely to survive long enough to give an infectious bite. Because zoophagy by itself often reduces malaria transmission, it is not obvious at first that the benefits of diversion to cows would be outweighed by the loss of mortality from encounters with human based interventions such as IRS in human dwellings. Our model results mean that while transmission by anthropophilic mosquitoes can be reduced to below the elimination threshold (*R*_*0*_ < 1) by increasing human-based interventions, transmission by zoophilic mosquitoes remains above the elimination threshold (*R*_*0*_ > 1) throughout the range of practicable intervention efficacy/coverage (Fig. 3, inset).

**Figure 4:**
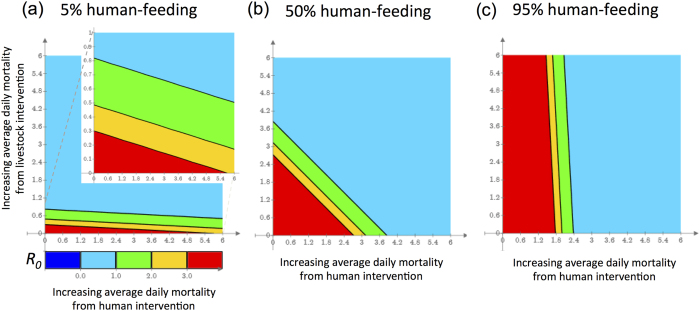
Contour plots showing *R*_*0*_ resulting from increases in average daily instantaneous mortality rate during feeding cycles involving attempted human (x axis) or livestock (y axis) feeding, arising from public health interventions. The blue area shows mortality combinations that push *R*_*0*_ below the eradication threshold. Plots are shown assuming (**a**) 5% of feeds are taken on humans, (**b**) 50% of feeds are taken on humans, (**c**) 95% of feeds are taken on humans.

The flip side of zoophily is that small increases in mortality associated with non-human feeding have greater impact on transmission than equivalent proportional increases in mortality associated with human feeding (Fig. 4). Moreover, while human-based interventions alone cannot eliminate malaria transmission by zoophilic vectors, increasing mortality in the zoophilic cycle generates substantial benefits in terms of reducing *R*_*0*_ below the elimination threshold. These effects suggest even a modest contribution of a cattle-based intervention (this could mean low coverage or relatively low efficacy in terms of engendered mortality) could tip the balance towards elimination (Fig. 4).

Our model considers feeding on only one host-type in each feeding cycle, and assumes that a feeding mosquito is only affected by interventions associated with the location of the chosen host. The field data show some mosquitoes taking both human and livestock blood feeds in one feeding cycle, and mosquitoes resting in different locations to their chosen hosts. Given that we define the proportion of human feeds as the proportion of feeding cycles that include a human blood meal, whether or not combined with a livestock feed, the model assumptions are conservative based on the field data. The model assumes that all human-fed mosquitoes, and no cattle-fed mosquitoes, are subject to incremental mortality from interventions applied to human dwellings, and conversely, that no human-fed mosquitoes and all cattle-fed mosquitoes are subject to the effects of cattle-shed based interventions. Since cattle-fed mosquitoes resting in human dwellings are substantially outnumbered in the field results by human-fed mosquitoes resting in cattle sheds, the model results will tend to overstate the impact of human-dwelling interventions and understate that of cattle-shed interventions on *R*_*0*_, making our conclusions robust to this model assumption in the context of the empirical study.

## Discussion

Our empirical work demonstrates that the key malaria mosquito vectors in our study area (which as indicated, is one of the highest transmission settings in India) are strongly zoophilic and exophilic. The *An. culicifacies* complex is generally considered zoophilic and our observation that adults tend to rest more often in cattle sheds than human dwellings, matches other research in this area^17^,^29^. We also found that *An. fluviatilis* was more commonly found resting in cattle sheds than human dwellings. This observation contrasts with previous studies in Sundergarh district, which showed *An. fluviatilis* to prefer to rest in human dwellings^13^,^17^. However, these earlier studies indicated a predominance (98%) of S-type, which is strongly anthropophilic^13^,^17^, whereas we found the majority of *An. fluviatilis* to be T-type, which is known to be more zoophilic and exophilic^13^,^30^. Other recent work in nearby Keonjhar district (also in Odisha state) similarly observed a high density of T-type relative to S-type (75.5% T and 9.4% S, remaining type U), reflecting a change compared with historical patterns^31^. Moreover, compared with previous studies, we found that even S-type *An. fluviatilis* were more abundant in cattle sheds than human dwellings. Thus, there appears to have been a shift both in species complex composition and additionally a change in S-type behavior towards zoophily/exophily, making their ecology more similar to T-type. We found relatively high levels of cattle feeding for both vector species, including the declining *An. fluviatilis* S-type, which suggests their recent shift to a more zoophagic feeding behavior could be adaptive in the remaining S-type population, possibly because they would be less exposed to traditional controls. This change from greater than 90% human blood feeding in S type^13^,^32^ to current zoophilic behaviors could indicate a response to the intensified use of IRS and LLINs within domestic dwellings over the last 10 years, mirroring changes in vector composition in certain parts of Africa from highly anthropophilic *An. gambiae* ss to zoophilic *An. arabiensis* that has occurred following wide scale deployment of LLINs^33^,^34^,^35^.

We also observed that mosquitoes fed exclusively on humans could be found resting in cattle sheds, and the converse was also true where mosquitoes fed exclusively on cows could be found resting in human dwellings. These data suggest regular movement of individual mosquitoes between indoor and outdoor feeding and resting habitats. Further evidence of interchange between hosts is provided by the relatively high levels of mixed human and bovine blood meals found in both vector species (e.g. of those found to have blood fed and collected from cattle sheds, 73.2% of *An. fluviatilis* T-type and 68.2% *An. culicifacies* BCE-type had mixed blood meals), suggesting that at least some individuals are feeding on humans and cattle during the same or consecutive nights^23^. Other studies have shown *An. culicifacies* B-type^36^ and *An. fluviatilis* T-type^17^ to have high levels of mixed feeding (though with the *An. fluviatilis* data, the sample size was very small). This behavior likely has important implications for transmission since multiple feeding within the same gonotrophic cycle increases the potential for human-vector contact^36^. One previous study conducted in neighboring Chhattisgarh State reported no mixed feeding for *An. fluviatilis* S or T-type^32^. These contrasting results could reflect local differences in feeding behavior but it is also possible that the gel electrophoresis method used to determine blood meal type in the earlier study is less sensitive than the PCR methods used here (and see also ref. 31).

Overall, the empirical data suggest that malaria transmission in this region of Odisha is sustained largely by zoophilic vectors. Our model analysis indicates that continued scale-up of conventional tools such as IRS or ITNs delivers diminishing returns and is unlikely to eliminate malaria in such settings. However, additional or redirected control effort towards the zoophilic cycle could be transformative.

One method to target zoophilic mosquitoes is to make the livestock themselves toxic to mosquitoes using either topical^37^,^38^ or systemic insecticides (endectocides) such as fipronil or ivermectin^39^,^40^,^41^. Treating individual cows might be logistically challenging, but previous studies have demonstrated reduced transmission using such an approach^38^.

Another strategy is to treat livestock structures in an equivalent way to conventional IRS. This strategy may offer additional benefits, given that anthropophagic mosquitoes appear to prefer to rest in cattle sheds even if they have fed on humans^42^ (Figs 2 and 3). IRS protects at the community level and impact is maximized when coverage is high. Historic research demonstrates that transmission by zoophilic vectors increases with greater adult mosquito densities^43^. Treating only human dwellings reduces effective coverage as cattle sheds provide an unsprayed refuge for mosquitoes (potentially the majority)^44^ (Fig. 4).

The choice of chemical for IRS in cattle shelters needs careful consideration. The most widely used chemicals for domestic IRS are pyrethroids and DDT and both exhibit repellency^4^,^45^. Repellency from the home is a good thing but for IRS in cattle shelters, there is the risk, though no evidence^46^, that repellency could potentially drive mosquitoes into human dwellings. There are numerous non-repellent actives including established products such as organophosphates (e.g. malathion and pirimiphos methyl) and carbamates (e.g. bendiocarb and propoxur), together with new classes of insecticides such as neonicotinoids (e.g. clothianidin). Further, combining the use of repellent products in domestic dwellings and non-repellent products in cattle shelters could create a ‘push-pull’ effect to enhance overall control^9^. Greater choice of actives could address issues of insecticide resistance in this region^47^,^48^, where *An. culicifacies* has been found resistant to all classes of chemicals used in some areas, although *An, fluviatilis* remains largely susceptible with the exception of some findings of DDT resistance outside of Odisha state. Greater choice of actives would also create valuable opportunities for the development and testing of novel insecticide resistance management strategies such as rotations or mosaics^49^.

Whether treatment of cattle shelters could select for behavioral resistance (i.e. changes in feeding and resting behavior towards anthropophily) is unclear. Given that even small increases in incremental mortality associated with zoophilic behavior can have a large impact, using low coverage could still be effective and should impose weak selection for increased anthropophily. Alternatively, ensuring that both cattle shelters and human dwellings are treated at high coverage with different classes of lethal chemical should limit capacity for any behavioral change (in a similar way to combination therapy, or any integrated vector management strategy with two modes of action)^50^. A study in India comparing IRS treatments in houses and cattle sheds with IRS treatments in houses alone, showed the combined treatment yielded the greatest reduction in malaria cases but found no evidence for behavioral change^51^.

The range of actives suitable for IRS in cattle shelters should be greater than conventional IRS, as the safety requirements that constrain product choice in human dwellings are likely to be less stringent for applications around livestock. Issues of end-user acceptance should also be relaxed. One study evaluating impact of DDT for IRS in India found refusal rates up to 70% due to factors such as decolorization, bad smell, increase in bed bug nuisance, and contamination of food grains^52^. The same study reported no objections to treatment of cattle shelters. Economic constraints might also be reduced, as the wider choice of possible actives could include cheaper products or formulations that enable less frequent spraying^53^. Common behaviors that affect persistence, such as replastering walls after treatment^52^, are also less likely to be a problem in cattle shelters. Importantly, the technology for implementing IRS in cattle shelters is proven^54^ and does not require years of further R&D before it can have an impact at scale.

## Conclusions

India has seen a substantial decline in malaria mortality and morbidity in recent years^55^, due in large part to the broad-scale use of IRS and ITNs in key transmission settings^56^. While this success is encouraging, our analysis indicates that sustained, or even incremented use of these tactics is unlikely to be sufficient to drive malaria over the edge in areas where zoophilic vectors dominate.

The historic control campaigns in India during the 1950’s and 1960’s were centered on IRS, with insecticides strategically applied to all structures where mosquitoes could feed and rest, including cattle shelters^57^. These control efforts took India to the brink of malaria eradication but ultimately faltered because of a complex of financial, logistic, and political factors^58^. When control efforts were reinvigorated at the turn of this century, the action plan for mosquito control consisted of IRS, ITNs, and some targeted larval control^59^,^60^. However, current policy restricts IRS to domestic dwellings^27^,^60^. The return to a more comprehensive approach that includes the treatment of cattle sheds with non-repellent chemicals (or other tools that explicitly target zoophilic behavior) could facilitate malaria elimination in India and elsewhere, where the majority of malaria mosquitoes are zoophilic^61^. That this could be done by potentially redirecting current efforts (i.e. potentially limited additional costs) and with products that can be used now (unlike numerous prospective control tools that are still far from operational use) is essential given the 2030 elimination target.

## Additional Information

**How to cite this article:** Waite, J. L. *et al*. Increasing the potential for malaria elimination by targeting zoophilic vectors. *Sci. Rep.* **7**, 40551; doi: 10.1038/srep40551 (2017).

**Publisher's note:** Springer Nature remains neutral with regard to jurisdictional claims in published maps and institutional affiliations.

## Supplementary Material

Supplementary Material

## Figures and Tables

**Table 1:** Parameter/variable definitions.

Standard *R*_*0*_ model	Description	Revised *R*_*0*_ Model
*m*	Total number of susceptible mosquitoes per person which will choose to feed on a human host	*m*_*z*_
	Total number of susceptible mosquitoes per person with human-related intervention in place	_*m0*_
	Proportion of feeding cycles in which a blood meal is taken on a human host.	*Z*
*a*	Bite rate per mosquito per day	*a*
*c*	Proportion of bites on infectious humans producing infection in mosquito.	*c*
*μ*_*2*_	Vector average per day instantaneous mortality rate (in the absence of interventions)	*μ*_*2*_
	Increase in the average instantaneous daily mortality rate during a feeding cycle for mosquitoes attempting to feed on a human, arising from human-related intervention, as a proportion of the rate in the absence of any intervention.	*Δ*_*H*_
	Increase in the average instantaneous daily mortality rate during a feeding cycle for mosquitoes attempting to feed on a non-human host, arising from cowshed-related intervention, as a proportion of the rate in the absence of any intervention.	*Δ*_*L*_
*b*	Probability that a bite from an infectious mosquito on a human host will generate a human *Plasmodium* infection.	*b*
*γ*	Per day instantaneous recovery rate in human host.	*γ*
*τ*	Time in days from acquisition of *Plasmodium* infection to infectiousness in vector.	*τ*

**Table 2:** Sibling species type composition of the *An. fluviatilis* and *An. culicifacies* complexes in the study area as revealed by allele specific PCR, showing their collection location, either human dwellings (HD) or cattle sheds (CS).

Mosquito species	Sibling species type	Total identified (sporozoite positive)	Relative percentage of type by species	Number in HD, number in CS
*An. culicifacies*	AD	4	0.2%	1, 3
*An. culicifacies*	BCE	1770 (2)	99.8%	95, 1675
*An. fluviatilis*	S	23	13.6%	3, 20
*An. fluviatilis*	T	146	86.4%	16, 130
*An. fluviatilis* (2^nd^ collection)	S	24	24.7%	3, 21
*An. fluviatilis* (2^nd^ collection)	T	73	75.5%	23, 50

*An. fluviatilis* types U and V were not found.
