# Supplementary material for: Increasing the potential for malaria elimination by targeting zoophilic vectors
Source: Sci Rep. 2017 Jan 16;7:40551. doi: 10.1038/srep40551 (PMC5238397; doi:10.1038/srep40551)
Supplement: Supplementary Material [file srep40551-s1.pdf]

Increasing the potential for malaria elimination by targeting zoophilic vectors

Jessica L. Waite, Sunita Swain, Penelope A. Lynch, S.K. Sharma, Mohammed Asrarul Haque, Jacqui Montgomery, Matthew B. Thomas

S1 Model derivation

The model is based on a standard expression for R_0

$$R_0 = \frac{ma^2bc}{\mu_2\gamma} e^{-\mu_2\tau_2}$$

(*Anderson and May eq 14.11 adjusted per text page 400 to reflect low human mortality rate relative to latent period*)

m = total number of *Plasmodium*-susceptible mosquitoes per person.
a = biting rate per mosquito per day.
c = proportion of bites on infectious humans producing infection in mosquito.
μ_2 = mosquito instantaneous background daily mortality rate.
b = probability that a bite from an infectious mosquito on a human host will generate a human *Plasmodium* infection.
γ = per day instantaneous recovery rate in human host.
τ = period from acquisition of *Plasmodium* infection to infectiousness in mosquito / days.

S2 Explanation of simple R0-based model for exploring the impact of zoophagy and interventions applied to livestock sheds on malaria transmission

Model Assumptions

The model considers as its baseline a vector population subject to lethal interventions applied via human dwellings and explores the effects of varying the probabilities of taking a human feed and the effects of adding cowshed-based interventions.

- Individual vectors are assumed to bite both human and livestock hosts with a given probability per feeding cycle of selecting a human host.
- Vectors are assumed to feed once per feeding cycle.
- Vector mortality and host choice is assumed to be unaffected by vector age.
- Once infectious, vectors do not recover and become non-infectious.
- Juvenile density dependence effects mean that changes to the adult vector population size do not affect the number of newly-mature adults joining the population per day.
- The human population size is not changed as a result of the interventions being considered

Parameter/variable definitions

m_0 = total number of susceptible mosquitoes per person with human-related intervention in place.
m_z = total number of susceptible mosquitoes per person which will choose to feed on a human host
Z = proportion of blood meals taken on humans.
a = biting rate per mosquito per day.
c = proportion of bites on infectious humans producing infection in mosquito.
μ_2 = mosquito instantaneous background daily mortality rate in absence of interventions.
b = probability that a bite from an infectious mosquito on a human host will generate a human *Plasmodium* infection.
γ = per day instantaneous recovery rate in human host.
τ = time in days from acquisition of *Plasmodium* infection to infectiousness in vector.
Δ_H = increase in the average instantaneous daily mortality rate during a feeding cycle for mosquitoes attempting to feed on a human, arising from human-related intervention, as a proportion of the rate in the absence of any intervention.
Δ_L = increase in the average instantaneous daily mortality rate during a feeding cycle for mosquitoes attempting to feed on a non-human host, arising from cowshed-related intervention, as a proportion of the rate in the absence of any intervention.

Given an adult vector population size in the presence of a given human-feeding related intervention, m_0, the introduction of a new source of mortality applicable to livestock-feeding vectors will change the adult population size. With an assumed constant rate of recruitment to the adult population, the average age structured survival probabilities of an individual vector also represent the age structure of the adult population.

The average per day mortality for the vector population before introducing the livestock-related intervention is $\mu_2\left(1-Z\right)+Z\mu_2\left(1+\Delta_H\right)=\mu_2\left(1+Z\Delta_H\right)$

The average per day probability of survival for the vector population before introducing the livestock-related intervention is therefore $e^{-\mu_2\left(1+Z\Delta_H\right)}$. The population size as a proportion of the number of adults joining the population per day is therefore $1+e^{-\mu_2\left(1+Z\Delta_H\right)}+\left(e^{-\mu_2\left(1+Z\Delta_H\right)}\right)^2+\left(e^{-\mu_2\left(1+Z\Delta_H\right)}\right)^3+......+\left(e^{-\mu_2\left(1+Z\Delta_H\right)}\right)^\infty$

which can be written as $\dfrac{1}{1-e^{-\mu_2\left(1+Z\Delta_H\right)}}$ using the standard sum for this infinite series.

After introducing the livestock-related intervention, a similar process gives a vector population size as a proportion of the number of adults joining the population per day of $\frac{1}{1-e^{-\mu_2(1+Z\Delta_H+(1-Z)\Delta_L)}}$.

With m_0 as the number of vectors per person with only the human-related intervention in place, and assuming that the human population size does not change, the number of vectors per person after introducing the livestock-related intervention will therefore be $m_0 \frac{\left(\frac{1}{1-e^{-\mu_2(1+Z\Delta_H+(1-Z)\Delta_L)}}\right)}{\left(\frac{1}{1-e^{-\mu_2(1+Z\Delta_H)}}\right)} = m_0 \frac{1-e^{-\mu_2(1+Z\Delta_H)}}{1-e^{-\mu_2(1+Z\Delta_H+(1-Z)\Delta_L)}}$

The human bite rate will be the vector bite rate × the proportion of bites targeting human hosts.

Standard R_0	Description	Revised R_0 Model
m	Number of mosquitoes per person	$m_0 \frac{1-e^{-\mu_2(1+Z\Delta_H)}}{1-e^{-\mu_2(1+Z\Delta_H+(1-Z)\Delta_L)}}$
a	Human bite rate per mosquito per day	aZ
μ_2	Vector average per day instantaneous mortality rate	$\mu_2\left(1+Z\Delta_H+(1-Z)\Delta_L\right)$

Substituting these expressions into the standard R_0 expression gives the equation shown in the main text,

$$R_0^{ZL} = m_0 \frac{1-e^{-\mu_2(1+Z\Delta_H)}}{1-e^{-\mu_2(1+Z\Delta_H+(1-Z)\Delta_L)}} \frac{a^2Z^2bce^{-\mu_2(1+Z\Delta_H+(1-Z)\Delta_L)\tau_2}}{\mu_2\left(1+Z\Delta_H+(1-Z)\Delta_L\right)\gamma}$$

S3 Supplementary materials, environmental data

Month	Avg. Rainfall mm
Oct-13	211.5
Nov-13	0
Dec-13	0
Jan-14	6.2
Feb-14	34.4
Mar-14	29.9
Apr-14	1.2
May-14	61.4
Jun-14	111.4
Jul-14	442.9
Aug-14	416.6
Sep-14	200.6

Months	Mean Temperature C
Oct-13	26.28
Nov-13	22.07
Dec-13	18.80
Jan-14	19.79
Feb-14	21.79
Mar-14	26.19
Apr-14	32.41
May-14	33.46
Jun-14	31.82
Jul-14	28.86
Aug-14	28.28
Sep-14	28.58

S4 Supplementary Material, Table S4

Table S4 *An. culicifacies* and *An. fluviatilis* sibling species types by collection location, showing the percentage of the overall catch with blood meal status and identities (if fed). 2nd coll. refers to the second collection period of *An. fluviatilis*. Percentages (rounded to two decimal places) refer to proportion of either *An. culicifacies* or *An. fluviatilis* found in each circumstance of location and feeding status by collection time. For example, all *An. culicifacies* add up to 100%, all *An. fluviatilis* from the first collection add up to 100%, as do all from the second collection.

Mosquito species	Sibling species type	Location HD= human dwelling, CS = cattle shed)	Bovine	Human	Mixed	Unfed	Other blood
An.culicifacies	AD	HD	0.00%	0.00%	0.06%	0.00%	0.00%
An.culicifacies	AD	CS	0.00%	0.00%	0.17%	0.00%	0.00%
An.culicifacies	BCE	HD	0.17%	2.48%	0.85%	1.86%	0.00%
An.culicifacies	BCE	CS	24.86%	11.44%	27.56%	26.04%	4.51%
An. fluviatilis	S	HD	0.00%	0.59%	0.59%	0.59%	0.00%
An. fluviatilis	S	CS	4.14%	5.33%	1.18%	1.18%	0.00%
An. fluviatilis	T	HD	2.96%	5.33%	0.00%	1.18%	0.00%
An. fluviatilis	T	CS	17.16%	4.37%	49.11%	3.55%	2.37%
An. fluviatilis	S	2nd coll. HD	0.00%	1.03%	1.03%	1.03%	0.00%
An. fluviatilis	S	2nd coll. CS	7.22%	9.28%	3.09%	2.06%	0.00%
An. fluviatilis	T	2nd coll. HD	11.34%	6.19%	3.09%	3.09%	0.00%
An. fluviatilis	T	2nd coll. CS	17.53%	5.15%	12.37%	16.49%	0.00%
